# Cannabidiol and Nano-Selenium Increase Microvascularization and Reduce Degenerative Changes in Superficial Breast Muscle in *C. perfringens*-Infected Chickens

**DOI:** 10.3390/ijms24010237

**Published:** 2022-12-23

**Authors:** Paweł Konieczka, Dominika Szkopek, Misza Kinsner, Paweł Kowalczyk, Monika Michalczuk, Damian Bień, Joanna Banach, Paulius Matusevičius, Joanna Bogucka

**Affiliations:** 1 Department of Animal Nutrition, The Kielanowski Institute of Animal Physiology and Nutrition, Polish Academy of Sciences, Instytucka 3, 05-110 Jabłonna, Poland; 2 Department of Poultry Science and Apiculture, University of Warmia and Mazury, Oczapowskiego 5, 10-718 Olsztyn, Poland; 3 Department of Animal Breeding, Institute of Animal Sciences, Warsaw University of Life Sciences, Ciszewskiego 8, 02-786 Warsaw, Poland; 4 Institute of Natural Fibres and Medicinal Plants-National Research Institute, Wojska Polskiego 71b, 60-630 Poznań, Poland; 5 Department of Animal Nutrition, Lithuanian University of Health Sciences, Tilzes 18, LT-47181 Kaunas, Lithuania; 6 The Independent Research Laboratory STANLAB LLC, Puchacza 1, 89-100 Nakło nad Notecią, Poland; 7 Department of Health Sciences, University of Bydgoszcz, Unii Lubelskiej 4C, 85-059 Bydgoszcz, Poland

**Keywords:** cannabis, selenium, necrotic enteritis, DNA damage, muscle abnormalities

## Abstract

Here, we demonstrated the potential of *Cannabis*-derived cannabidiol (CBD) and nanosized selenium (nano-Se) for the modulation of microvascularization and muscle fiber lesions in superficial breast muscle in *C. perfringens*-challenged chickens. The administration of CBD resulted in a decreased number of atrophic fibers (3.13 vs. 1.13/1.5 mm^2^) compared with the control, whereas nano-Se or both substances resulted in a decreased split fiber number (4.13 vs. 1.55/1.5 mm^2^) and in a lower number of necrotic myofibers (2.38 vs. 0.69/1.5 mm^2^) in breast muscle than the positive control. There was a significantly higher number of capillary vessels in chickens in the CBD+Nano-Se group than in the control and positive control groups (1.31 vs. 0.97 and 0.98, respectively). Feeding birds experimental diets lowered the activity of DNA damage repair enzymes, including 3,N4-ethenodeoxycytosine (by 39.6%), 1,N6-ethenodeoxyadenosine (by 37.5%), 8-oxo-guanine (by 36.2%), formamidopyrimidine (fapy)-DNA glycosylase (by 56.2%) and human alkyl adenine DNA glycosylase (by 40.2%) in the ileal mucosa, but it did not compromise the blood mitochondrial oxygen consumption rate (−2.67 OD/min on average). These findings indicate a potential link between gut mucosa condition and histopathological changes in superficial pectoral muscle under induced inflammation and show the ameliorative effect of CBD and nano-Se in this cross-talk due to their protection from mucosal DNA damage.

## 1. Introduction

Necrotic enteritis (NE), caused by the anaerobic bacterium *Clostridium perfringens* (or *C. perfringens*), is responsible for major economic losses in the poultry industry due to impaired bird performance and increased morbidity and mortality rates [1]. Due to the ban on the preventive use of antibiotics and antibiotic growth promoters in the EU (Regulation (EC) No. 1831/2003), health problems have arisen in broiler chicken flocks, including an outbreak of *Clostridium perfringens* infections. The acute form of the disease leads to increased mortality in broiler flocks [2]. In the subclinical form, there is damage to the intestinal mucosa caused by *C. perfringens*, leading to reduced digestion and absorption and consequently to lower body weight gain and increased feed conversion ratio. Importantly, *C. perfringens* in poultry poses a risk of transmission to humans through the food chain [3].

In recent years, the poultry industry has made significant progress in breeding broiler chickens, resulting in shorter fattening times and faster weight gain. However, the rapid growth of birds results in histological and biochemical modifications of the muscle tissue, the occurrence of myopathies, and ischemic and degenerative changes in the muscles [4]. Histopathological lesions in myopathies include necrosis and atrophy of fibers, split fibers, giant fibers and connective tissue hyperplasia [5].

With the aim of minimizing the consumption of pharmaceuticals, possibilities have been sought to replace drugs with health-promoting preparations that are not medicines but exert similar effects. One bioactive substance that has been looked at in great detail is cannabidiol (CBD). Research on CBD has discovered a new, so-called endocannabinoid system, which regulates not only brain function but also the immune system. Studies examining the effects of cannabinoid-based drugs on immunity have shown that many cellular and cytokine mechanisms are suppressed by these agents [6]. This leads to the hypothesis that the aforementioned drugs may be of value in the treatment of chronic inflammatory diseases [7]. The mechanism underlying CBD action has not yet been fully elucidated, but it may represent biological activity in the regulation of inflammation through close affinity to the processes mediating necrosis and intestinal inflammation in chickens. Since NE is a serious problem in the poultry industry, CBD could be an interesting solution in prevention strategies. Therefore, additives that can help to improve birds’ health while maintaining performance indicators are currently of great interest [8]. While it is overall consensus that the abuse of the most abundant bioactive substance found in *Cannabis* plants—tetrahydrocannabinol—is usually associated with toxicity to the host, current findings indicate that CBD, the second active compound in *cannabis* plants in terms of abundance (but without psychedelic effects on the host), is one of the most promising cannabinoids [9,10]. On the other hand, reports indicating CBD toxicity based on preclinical and clinical studies, as well as experiments involving animals and in vitro models, also exist [11]. More recently, an increasing body of evidence obtained using different models has suggested that CBD exposure (depending on dose) may affect the mitochondrial respiration efficiency of immune cells as well as mitochondrial fission and fusion, which are of key importance during response to high metabolic stress [12].

Selenium (Se) is another bioactive substance that has caught the attention of poultry producers. Selenium has been recognized as an essential trace element to animals and humans that is actively involved in oxidative stress resistance, reproductive performance and immune function [13]. Selenium is an important antioxidant mineral in animals and is known to affect feather production and maintain cellular integrity in bird tissues. Various Se forms are available for supplementation in animal and poultry feeds, e.g., inorganic sodium selenate (Na_2_SeO_4_) and sodium selenite (Na_2_SeO_3_) [14,15]. The organic form, such as that of selenized yeast, is similar to Se compounds found in cereals and feeds. However, selenium concentrations in feed ingredients vary widely depending on the plant species and, in particular, the Se status of the soil. Therefore, Se supplementation is necessary in poultry nutrition to provide a safety margin against its deficiency and to maintain production efficiency [16]. With the development of nanotechnology, the effect of particles in “nano” form has begun to be studied. Nanoscale selenium is of great interest as a food additive, especially for individuals with selenium deficiency, but also as a therapeutic agent without significant side effects in medicine. The nano form of selenium has attracted even more attention due to its high bioavailability and low toxicity compared with inorganic and organic forms, where inorganic compounds are more toxic than organic ones [17]. Studies have demonstrated the antimicrobial and antifungal activity of nano-Se [18]. In the context of potential bioactivity/toxicity of Se, it was evidenced, using Lilly Laboratories culture-Porcine Kidney 1 cell lines, that Se produced significant protection against Cd-induced apoptosis by mediating oxidative stress, which in turn had ameliorative effects on mitochondrial dysfunction [19]. In another study, it was shown that Se was effective in the prevention of mitochondrial damage caused by Adriamycin in Sprague–Dawley rats [20]. Eventually, Se nanoparticles were used to investigate their potential in malignant ascites treatment using a mouse model. It was revealed that Se could influence mitochondrial function and induce cell apoptosis [21]. Taken together, the results suggests that both molecules (CBD and nano-Se) have a close affinity to mitochondrial function; thus, based on this interaction, their beneficial/toxic effects on the host can be assessed. Nano-Se can be obtained via chemical synthesis, physical methods or even in a biological way (green synthesis), using microorganisms or plant extracts, which indeed determine their biological action to a high extent [22]. Among others, nano-Se obtained via chemical synthesis seems to be the most suitable component for nutritional applications, because in this form, it is positively charged, biocompatible, non-immunogenic, nontoxic, pH sensitive and biodegradable [22].

To date, research has focused on the effects of cannabidiol and nano-selenium on the improvement of intestinal barrier function and bacterial enzyme activity in chickens. This study investigated the bioactive properties of CBD and nano-Se in broilers with potential beneficial effects on gut health and function. Konieczka et al. [8] found that CBD and nano-Se could modulate the response of chickens to *C. perfringens* infection, which in turn may provide time for effective intervention. The beneficial effects of both agents on host physiology were manifested in supporting intestinal barrier function through increased expression of genes that control intestinal integrity (tight junction proteins; TJPs). CBD and nano-Se promoted changes in extracellular bacterial enzyme activity toward increased energy uptake in challenged chickens but showed no counter-effects on mediating the host response to infection. Recently, an increasing number of studies have indicated a close relationship between intestine condition and processes determining the physicochemical properties of meat in poultry [23,24,25]. The metabolism of key elements, including peptides, fatty acids and amino acids, in the host depends to a high extend on gut barrier function [26]. As reported, both CBD and nano-Se manifest direct and indirect actions on the same mechanisms of the gut physiological status, particularly the modulation of gut bacterial composition and activities; thus, we speculated that both CBD and nano-Se could be more effective in supporting gut integrity and should thus affect meat properties, especially in challenged birds, as this condition leads to fermentation disturbance, as proven in various models [27,28,29]. For instance, chickens challenged with *C. perfringens* were shown to have a higher abundance of several foodborne pathogens in the gut, including *C. jejuni*, *E. coli* and *L. monocylogenes*; this treatment also affected the transcript levels of many genes regulating host metabolism [30]. In our previous study [31], in a turkey model, we found that challenging birds with *C. perfringens* had a significant effect on the sarcoplasmic protein profile of the breast muscle, indicating a strong association between *C. perfringens* infection and the function of glycolytic enzymes in turkeys, which could lead to significant consequences in cell metabolism [32]. In line with this, [33] showed a possible linkage between reduced vessel density and ultrastructural alterations in chicken breast muscle in early-stage wooden breast myopathy development, and the enlargement of the sarcoplasmic reticulum and greater severity of mitochondrial morphological alterations due to osmotic imbalance. The authors of [34] reported that skeletal muscles, including breast muscle, in chickens and turkeys can be affected by *C. perfringens* due to immune system failure caused by different stressors. In another report [35], it was shown that *C. perfringens* impairs the muscle regeneration process due to induced necrosis via the disturbance of collagen deposition in injured tissue, alternations in capillary vessels and nerves in infected muscle, as well as changes in the transcript levels of gene mediators of the inflammatory response and fibrosis in infected muscle. Both CBD and nano-Se may have the potential to interact with all of the mentioned pathogenesis factors. 

However, the most significant correlation between host intestinal response and meat quality in chickens was found in our recent study, in which dietary CBD in *C. perfringens*-infected chickens reduced meat volatile compound levels correlated with bacterial activity [36]. In the latter study, we showed that the only group of birds with a different VOC profile (including spoilage markers) was the *C. perfringens*-challenged group, indicating a strong impact of *C. perfringens* infection on meat properties. This could be attributed to the fact that *C. perfringens* affects lipid metabolism by downregulating the expression of fatty acid catabolism-related genes, including peroxisome proliferator-activated receptor-alpha, carnitine palmitoyl transferase 1 and acyl CoA oxidase 1 [37], which play important roles in lipid metabolism and thus strongly contribute to meat sensory properties. Eventually, Bień et al. [38] demonstrated that using different forms of selenium, including the nanosized form, in chicken diet was effective in the modulation of the fatty acid profile, and lipid and enzymatic indices of fatty acid metabolism in breast muscle as well as in the liver in birds.

Corresponding to the above reported findings, the aim of this study was to verify whether the use of CBD and Se in the form of nanoparticles in the diet of broiler chickens subjected to necrotic enteritis caused by *Clostridium perfringens* affected the degree of blood supply to the superficial breast muscle and the extent of pathological changes. At the same time, the study verified whether the use of the tested substances in the diet of chickens exerted toxic effects on selected markers of intestinal barrier function and mitochondria.

## 2. Results

### 2.1. Breast Meat Microstructure in Response to Treatment

There was a significantly higher number of capillary vessels in the muscle of chickens in the CBD + Nano-Se group than in CON and CON positive birds (*p* < 0.05) (Table 1). At the time of CBD and nano-Se administration to the chickens, a tendency of improved microvascularity of the muscle was observed. A lower number of necrotic myofibers was found in the chicken groups infected with *C. perfringens* after the addition of both bioactive substances, as well as selenium nanoparticles alone, compared with the CON positive group. The smallest number of atrophic fibers was observed in the muscle of CBD group birds compared with CON. Noteworthy is the number of split fibers, as a significant increase in the number of these lesions was observed after infecting chickens with *C. perfringens* compared with the control group. The administration of CBD, nano-Se and CBD + nano-Se reduced the extent of splitting compared with the CON positive group (*p* < 0.05). There were no differences in the content of giant fibers nor the thickness and number of fibers among the studied groups of birds (Table 1).

### 2.2. Gut Barrier Condition as Response to Treatment

**Figure 1:**
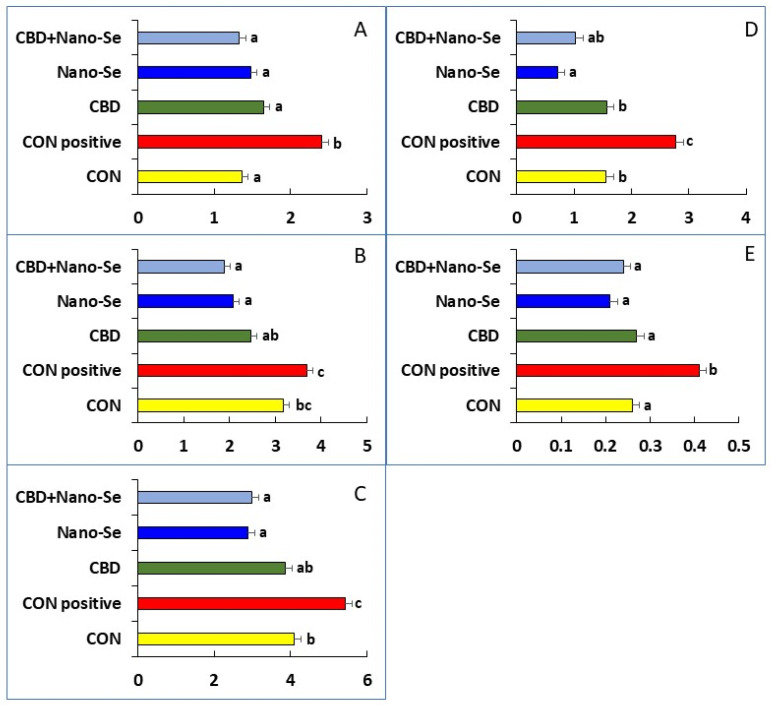
Excision activity (pmol/h/mg protein) of 3,N4-ethenodeoxycytosine (**A**), 1,N6- ethenodeoxyadenosine (**B**), 8-oxo-guanine (**C**), formamidopyrimidine (fapy)-DNA glycosylase (**D**) and human alkyl adenine DNA glycosylase (**E**). CON—untreated control group; CON positive—birds fed a CON diet challenged with *Clostridium perfringens* on days 15, 16, 17 and 18 of age; CBD—CON positive + dietary supplementation with cannabidiol; Nano-Se—CON positive + dietary supplementation with nano form of selenium; CBD + Nano-Se—CON positive + dietary supplementation with both additives. ^a,b,c^ Different letters represent significant differences (*p* < 0.001). Error bars represent mean standard error values for 8 birds in each dietary treatment.

The activity of DNA damage repair enzymes in the ileum is shown in Figure 1. etC repair activity was significantly higher in the CON positive group than in the other groups (*p* < 0.001). With respect to the repair activity of the etA enzyme, no significant differences were recorded between the CON and CON positive groups, and both groups showed higher enzyme activity than the Nano-Se and CBD + Nano-Se groups, with activity being higher in CON positive than in the CBD group (*p* < 0.001). The activity of the 8oxodG enzyme was the highest in the CON positive group and did not differ significantly between CON and CBD, while it was the lowest in the Nano-Se and CBD + Nano-Se groups (*p* < 0.001). Fpg activity was the highest in the CON positive group and did not differ significantly among the CON, CBD and CBD + Nano-Se groups, and it was the lowest in the Nano-Se group (*p <* 0.001). The activity of hAAG was significantly lower in the other groups compared with the CON positive group (*p* < 0.001). 

### 2.3. Oxygen Consumption Rate in Platelet Mitochondria as a Response to Treatments

**Figure 2:**
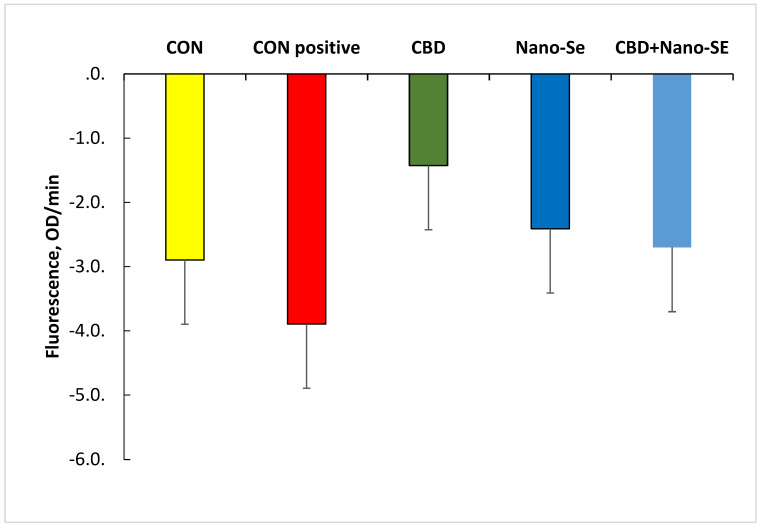
Oxygen consumption rate in platelet mitochondria expressed as the rate of fluorescence signal change per minute. CON—untreated control group; CON positive—birds fed a CON diet and challenged with *Clostridium perfringens* on days 15, 16, 17 and 18 of age; CBD—CON positive + dietary supplementation with cannabidiol; Nano-Se—CON positive + dietary supplementation with nano form of selenium; CBD + Nano-Se—CON positive + dietary supplementation with both additives. Error bars represent the mean standard errors for 8 birds in each dietary treatment (SEM = 0.318 and *p* = 0.250).

The rate of oxygen consumption in platelet mitochondria, expressed as a change in fluorescence signal over time (intensity inversely proportional to the level of extracellular oxygen), is shown in Figure 2. The results indicate that neither dietary treatments nor *C. perfringens* challenge significantly affected the oxygen use of platelet mitochondria (*p* > 0.05).

## 3. Discussion

The available literature indicates a positive effect of both CBD [39,40,41] and nano-Se [14,16,42,43] on the quality of broiler chicken meat. Studies in rats show that CBD can provide moderate analgesic and anti-inflammatory effects without suppressing muscle recovery [44]. Konieczka et al. [8] investigated the activity of cannabidiol derived from *Cannabis sativa* and selenium nanoparticles in modulating the host response to challenge with *Clostridium perfringens* in broiler chickens under mild infection conditions. It should be noted that the infected chickens showed no clinical signs, confirming the potential risk of pathogen transmission into the food chain in the commercial sector. However, both CBD and nano-Se had a positive effect on chickens’ response to *C. perfringens*. The beneficial effect of both agents was manifested in the increased expression of genes determining intestinal barrier function. Both CBD and nano-Se promoted changes in gut bacterial enzyme activity to increase energy intake in challenged chickens and enhance potential collagenase activity. The results of the cited studies prompted the authors to conduct research concerning the effect of CBD and nano-Se on the microstructure of the breast muscle of chickens after infection with *C. perfringens*. There were no differences among the chicken groups in both the diameter and the number of muscle fibers, which was also confirmed by previous studies with other bioactive substances administered in feed and in ovo [5,45,46,47]. However, differences in muscle blood supply and changes in its microstructure were observed. There was a tendency of an increasing number of capillaries after CBD and nano-Se administration, and a significant improvement in blood supply was observed after supplying both substances simultaneously. This was also reflected in the number of pathological changes in the muscle. After the addition of selenium nanoparticles, as well as both bioactive substances concurrently, decreased muscle fiber necrosis was observed compared with the CON positive group. Pelyhe and Mézes [48] emphasized the role of selenium in antioxidant defense systems, as well as the prevention of cell damage in farm animals, which might explain the lowest number of necrotic fibers in the selenium-supplemented group. As reported by Bilgili and Hess [49], and Velleman [50], necrosis is one of the degenerative lesions primarily caused by limited blood supply to the muscle fiber, which may be associated with fewer capillaries and limited angiogenesis. In addition, the administration of cannabidiol (CBD group) resulted in a small number of atrophic fibers in the muscle compared with CON. As is well known, CBD is a phytochemical that shows a strong potential in the control of neurodegenerative disorders, which are the main cause of muscle fiber atrophy [51]. However, the number of split fibers deserves attention, as the administration of CBD, nano-Se and CBD + nano-Se resulted in decreased fiber splitting compared with the CON positive group (*p* <0.05) (Table 1). Fiber splitting is a lesion that requires special attention in the muscles of fast-growing chickens. The longitudinal splitting of muscle fibers is one of the degenerative changes that may be identified in transverse sections of muscle tissue. McRae et al. [52] reported that this lesion occurs in chickens showing myopathic changes as a result of metabolic stress associated with, among others, the functioning of larger fibers. The study by Bogucka et al. [5,45] also showed a positive effect of probiotics and synbiotics as additives in the nutrition of broiler chickens on reducing the degree of muscle fiber splitting, which indicated a significant relationships between the environment of the digestive tract and the physicochemical properties of breast muscle in chickens.

In the present experiment, the activity of DNA repair enzymes was studied as a marker of intestinal barrier condition in relation to dietary treatments. Since DNA repair is an important risk factor in the etiology of pathogen challenge, it may also serve as a good indicator of the effectiveness of the treatment efficacy of test substances [53]. One of the important modulators of DNA changes is oxidative stress resulting from the increased sensitivity of cells to oxidizing and alkylating agents, which is associated with incomplete repair of single-strand breaks [54]. To the best of our knowledge, there are no data in the literature regarding changes in the activity of DNA repair enzymes in chickens infected with *C. perfringens*; however, in studies in a rat model challenged with lipopolysaccharide from S. typhimurium and E. coli, an increased rate of oxidative DNA damage was found to be originated by either a direct attack of ROS on DNA (8-oxoG) or the adduction of lipid peroxidation products (εA and εC), as well as a concomitant increase in DNA repair enzyme activity [55]. For this reason, it can be assumed that they are good markers of the effectiveness of treatments. Indeed, in our experiment, the repair activity of all enzymes except for etA was the highest in the CON positive group, indicating an increase in DNA damage in the ileum caused by *C. perfringens* compared with the non-infected group. In contrast, the repair activity in the experimental groups was significantly lower or did not differ from that in the CON group, suggesting an ameliorating effect of CBD and nano-Se on DNA damage in intestinal epithelial cells under *C. perfringens* challenge conditions. We speculated that both bioactive substances (CBD and nano-Se) could improve the integrity and function of the ileal barrier, as in a previous study, we showed that both additives increased the expression levels of TJP genes, including GLP-2, JAM-2, ZO-1 and TLR-4, in the intestines of *C. perfringens*-infected chickens [8]. A number of cases has indicated an association between stress-regulating factors of the host response and meat features, including myopathies such as white striping, wooden breast and spaghetti meat, which are associated with breast muscle vascularization and oxygen supply [56]. A report by Pampouille et al. [57] demonstrated that broiler meat disorders are associated with histological changes or transcript levels of oxygen carrier genes. Avian breast muscle disorders have also been linked to genes regulating the transition from the glycolysis pathway towards amino acid catabolism and lipid oxidation for the production of energy [58] and of long- medium-chain and monounsaturated fatty acids, as well as lipid metabolism [59,60]. Although these mechanisms are complex and require further research, the present study contributes to research by reporting the fact that meat vacuolization is affected by intestinal barrier function. 

Here, we used an oxygen consumption assay to investigate whether the bioactive agents used (CBD and/or nano-Se) exerted a toxic effect. Oxygen consumption assays allow respiration rates to be determined for the metabolic characterization and assessment of the toxic effects of treatments on mitochondrial function [61]. Lower extracellular oxygen levels indicate a higher rate of oxygen consumption in the mitochondria. Since mitochondria play a key role in the metabolic processes of the entire biological system, their impairment is the main mechanism of drug-induced toxicity, as has been demonstrated in various models [62,63,64]. In a recent study, we measured the rate of oxygen consumption in platelet mitochondria as an indicator of treatment effect, as this may have implications for further applications. Blood analysis is an easy-to-use approach; it can, therefore, be used to assess the effects of dietary treatment in birds [65]. Our findings indicated that neither CBD nor nano-Se (or their concurrent application) significantly affected mitochondrial function, as assessed in terms of oxygen consumption. Thus, it can be speculated that oral administration of CBD and nano-Se did not induce toxicity in birds at the doses tested under both optimal conditions and *C. perfringens*-induced inflammation; in particular, it did not impair platelet function during the inflammatory response to stress stimuli, and it has been suggested that platelets play a key role during antigen presentation [66,67].

## 4. Materials and Methods

### 4.1. Chemical Composition of Hemp Extract and Nano-Selenium

Hemp (*Cannabis sativa*) panicles were extracted from plants harvested in 2019 at Institute of Natural Fibers and Medicinal Plants in Poznań, Poland. Plants for the experiment were cultivated from certified seeds, and all procedures, including either plant cultivation or the collection of plant material, complied with institutional, national and international guidelines and legislation. The plants that were used for extract preparation were EU-registered (Research Centre for Cultivation Testing, Polish National List of Variety (NLI); Tygra No. R1865) and had a cannabinoid content below 0.2%. The plant material was collected, cut and dried at room temperature. The hemp extract was obtained at Supercritical Extraction Department, Institute of New Syntheses Institute, Puławy. Extraction parameters: pressure, 250 bar; temperature, 60 °C; flow rate, 40 kg CO_2_/kg hemp. Hemp extract contained 12% CBD, 0.49% tetrahydrocannabinol and 0.38% tetrahydrocannabinolic acid. The plants used to prepare extracts were registered in the EU (Research Centre for Cultivation Testing, Polish National List of Variety (NLI); Tygra No. R1865) and had a cannabinoid content of less than 0.2%. In the present experiment, the desired result of extraction was to obtain the highest possible concentration of CBD in the extract, which in consequence increased the content of other cannabinoids in the final extract, including tetrahydrocannabinol. Therefore, considering the inclusion level (15 g/kg diet) of CBD extract in the diet, the final concentrations were 0.0735 g of tetrahydrocannabinol per 1 kg feed and 1.8 g of CBD per 1 kg feed.

Nano-Se obtained through chemical synthesis was used in the form of a nanopowder with an average particle size of 10–45 nm, specific surface area (SSA) of about 30–50 m^2^/g and purity of 99.9%, as declared by the manufacturer (American Elements, Los Angeles, CA, USA).

### 4.2. Birds, Treatments and Challenge Model

A total of 360 birds (male broiler chickens of the Ross 308 line), reared from day 1 to 35, were used in the experiment. Chickens, from the first day of rearing, were placed in collective cages (9 individuals per cage) taking into account the average body weight. The study design included 5 experimental groups, with 8 replicates (cages) in each group—9 birds in each replicate. Chickens were fed a complete feed mix for broilers divided into 3 feeding periods, starter (days 1 to 7), grower (days 8 to 35) and finisher (days 36 to 42), according to the Ross 308 chicken feeding recommendations [68]. The experiment used CBD extract obtained from industrial hemp (*Cannabis sativa*) and/or nano-Se, which were added to the experimental diets (Table 2).

Birds from groups CON positive, CBD, Nano-Se and CBD+Nano-Se were infected with *Clostridium perfringens* on days 15, 16, 17 and 18, according to the methodology reported previously [8]. Briefly, at 15, 16, 17 and 18 days of age, birds were challenged with 1 mL (per os, directly into the crop using a probe) of a *C. perfringens* overnight culture inoculum, freshly prepared for each subsequent day; bacteria were cultivated anaerobically in sterile brain heart infusion broth medium (Sigma-Aldrich) containing beef heart (5 g/L), calf brain (12.5 g/L), disodium hydrogen phosphate (2.5 g/L), D (+)-glucose (2 g/L), peptone (10 g/L) and sodium chloride (5 g/L) at the temperature of 37 °C and were maintained overnight (14 h) in a jar containing an atmosphere of 10% CO2, 10% H2 and 80% N2. *C. perfringens* inoculum was confirmed according to ISO standard 7937:2005 (PN EN ISO 7937, 2005) and contained approximately 10^8^ CFU/mL of *C. perfringens* type A strain 56 according to a pre-validated protocol [69], while chickens in the non-challenged group received the same dose of sterilized broth medium. Before *C. perfringens* challenge, 1 mL of coccidial cocktail containing *Eimeria* (E) species (*E. acervulina*, (5000 oocytes), *E. maxima* (3500 oocytes), *E. mitis* (5000 oocytes), *E. praecox* (5000 oocytes) and *E. tenella* (5000 oocytes)) was administered per os to all birds at 14 and 15 days of age to create a favorable intestinal environment [70] for the development of mild necrotic enteritis caused by *C. perfringens* colonization (Laboratorios HIPRA S.A., Spain). The chicken basal diet was composed of wheat (50.76%), soybean meal (21.76%), triticale (15.54%), fish meal (5.18%) and oilseed rape meal (4.15%) to create a favorable environment in the digestive tract for *C. perfringens* proliferation. The severity of infection in the intestinal tissue was assessed by a veterinarian based on necrotic lesions typical of *C. perfringens*. None of the birds showed severe necrosis, which is usually manifested by confluent necrosis of the mucosa of large parts of the gut segments, collapse of the intestinal lumen, lack of turgor, thin and fragile intestinal wall, advanced necrosis of the gut mucosa, and visible multifocal hemorrhage in various regions [71,72]. The experimental diets were supplemented with CBD at a level of 15 g/kg feed, while nano-Se was added in the amount sufficient to provide 0.3 mg Se/kg feed.

### 4.3. Evaluation of the Microstructure of the Breast Muscles of Broiler Chickens

**Figure 3:**
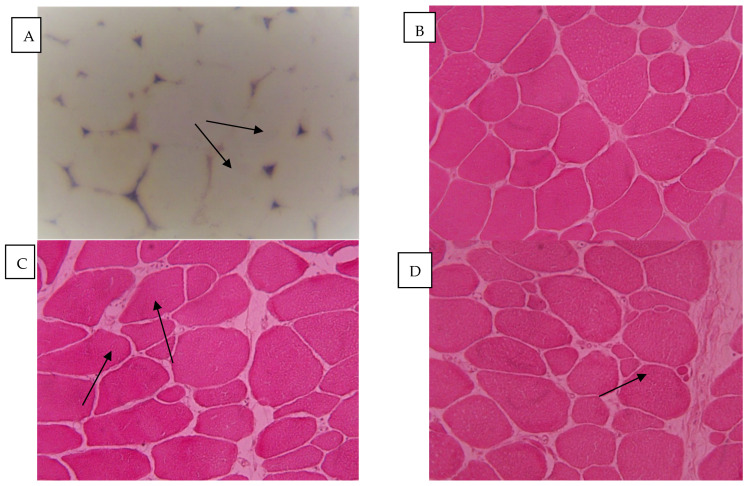

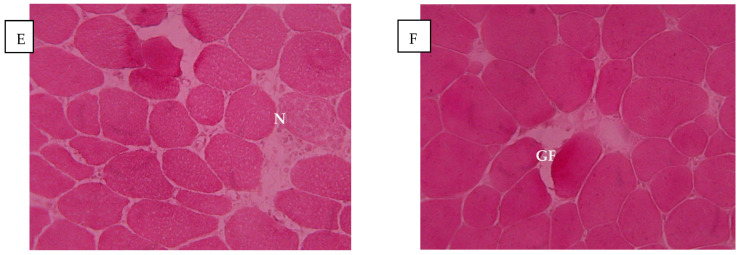
(**A**) Capillaries (arrows); alkaline phosphatase staining; magnification ×200. (**B**) Normal structure of superficial breast muscle. (**C**) Split fibers (arrows). (**D**) Atrophic fibers (arrow). (**E**) Necrosis (N). (**E**) Giant fiber (GF). (**B**–**F**) hematoxylin and eosin (HE) staining; magnification ×200.

Breast muscle samples were collected from chickens at 35 days of age (*n* = 8/group). The histological preparations that were used to evaluate the microstructure of broiler chicken breast muscles were prepared using the freezing technique. After fixation in liquid nitrogen (−196 °C), superficial breast muscle samples were cut in a cryostat (Thermo Shandon/Thermo Fisher Scientific, UK) into 10 µm thick sections. The thus-prepared sections were transferred onto slides and subjected to hematoxylin and eosin (HE) staining and reaction to alkaline phosphatase present in capillary endothelium. HE staining was used to measure muscle fiber diameter and determine the number of normal and altered fibers. The number of lesions such as necrotic fibers, atrophic fibers, split fibers and giant fibers was calculated. The alkaline phosphatase method was used to determine the number of capillaries in the studied muscle. Ten fields of view with the highest number of vessels, the so-called “hot spots”, were analyzed; subsequently, the number of capillaries per 1 muscle fiber was calculated (Figure 3). A Nikon Eclipse Ci microscope, equipped with a Delta Optical DLT-Cam PRO 6.3MP camera and DLT Cam Viewer software, was used to analyze histological images. Muscle analysis was performed on an area of 1.5 mm^2^.

### 4.4. Assessment of the Activity of DNA Damage Repair Enzymes in the Ileum

**Scheme 1:**
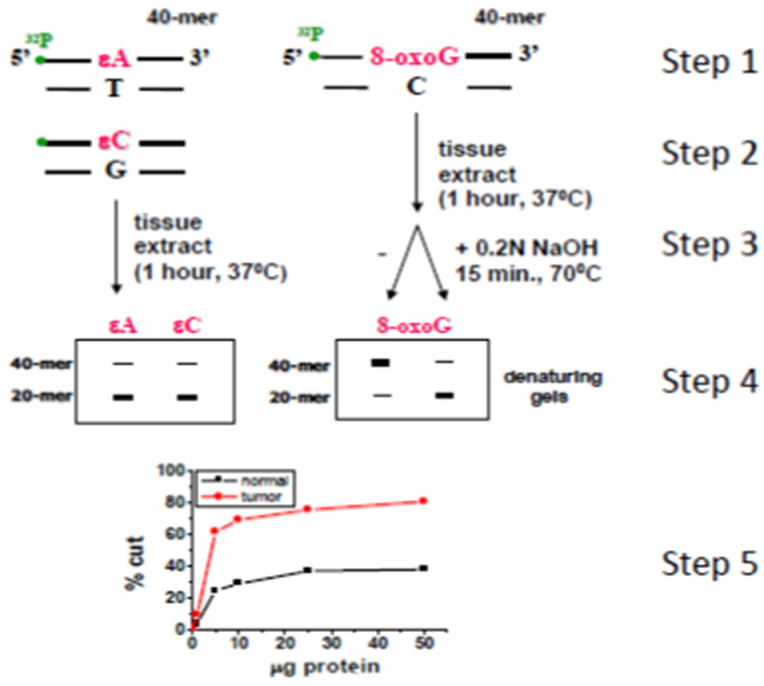
The nicking assay involves 5 main steps: 1—labeling of oligodeoxynucleotides containing εA, εC and 8-oxoG; 2—hybridization; 3—excision of εA, εC and 8-oxoG from oligonucleotides performed by glycosylases present in tissue extracts; 4—separation of reaction products; 5—detection and quantification of the reaction products. The resulting reaction products were analyzed using Imane Quant 5.2 and Microcal Origin programs for reaction product quantification. The cleavage percentage of a known amount of ³²P-labeled oligonucleotides relative to the total content was evaluated and expressed as picomoles of product/hour/microgram of protein.

The condition of the intestinal barrier was assessed using the repair activity assay and nicking assay method. On day 23 of age (5 days after the last *C. perfringens* challenge), 8 birds from each treatment were sacrificed, and ileal samples were collected and fixed. Briefly, the condition of the intestinal barrier was assessed on the basis of the repair activity assay with the nicking assay method [55,73] using oligonucleotides (40-mers) containing a single 3,*N*^4^-ethenodeoxycytosine (εC), 1,N6-ethenodeoxyadenosine (εA) and 8-oxo-guanine (8-oxoG) at position 20 in the sequence 5′-d(GCT ACC TAC CTA GCG ACC TXC GAC TGT CCC ACT GCT CGA)-3′. Oligonucleotides were obtained from Eurogentec Herstal (Herstal, Belgium) or Genset Oligos (Paris, France). Understanding the genotoxic properties of endogenous DNA damage as etheno-DNA adducts, such as 1,*N*^6^-ethenoadenine (εA), 3,*N*^4^-ethenocytosine (εC), *N*^2^,3-ethenoguanine (εG) and 1,*N*^2^-ethenoguanine (εG) and repair pathways, resulting in oxidative stress response, is fundamental to understand the mechanisms of diseases associated with chronic inflammation such as cancer, neurodegenerative diseases and aging. Due to the large range and pleiotropic effects of these products, knowledge about their molecular mechanisms of action is still fragmentary [55,73]. The principle of the nicking assay is based on the cleavage of an oligodeoxynucleotide at the site of modified bases (exocyclic DNA base adducts) such as etenoadenine (εA), etenocytosine (εC) and etenoguanine (εG) performed by glycosylases and AP-endonucleases present in tissue homogenates. The assay monitors the excision of εAde, εCyt and 8oxoG from a 5′-radiolabeled or phosphorescent synthetic DNA oligodeoxynucleotide performed by DNA glycosylases contained in tissue extracts, as well as the incision of abasic sites performed by AP endonucleases such as formamidopyrimidine (fapy)-DNA glycosylase (Fpg) and human alkyl adenine DNA glycosylase (hAAG) (Scheme 1).

### 4.5. Analysis of Mitochondrial Oxygen Metabolism Using the Oxygen Consumption Assay 

The rate of oxygen consumption in platelet mitochondria was assessed according to the previously described protocol [65]. Briefly, whole blood samples (2 mL) were collected from birds at 23 days of age (8 birds per group) into heparinized tubes. Blood was subsequently gently mixed to avoid microsphere formation, which could reduce the number of platelets and impair the results, and was placed on ice for 2 h to allow the sedimentation of individual blood cells to be achieved. Once a visible phase boundary was established, approximately 150 μL of the upper layer containing the platelets was collected and transferred to new tubes containing 300 μL of 10 mM dimethyl sulfoxide (DMSO) for cryopreservation. Samples were stored at −20 °C for further analyses. Before the analyses, fixed platelets were centrifuged at 3500× *g* for 5 min at 2 °C to remove DMSO. Isolated platelets were dissolved in 300 μL of HEPES-buffered Tyrode’s solution (119 mM NaCl, 5 mM KCl, 25 mM HEPES buffer, 2 mM CaCl_2_, 2 mM MgCl_2_ and 6 g/L glucose, adjusted with NaOH to final pH of 7.4). The extracellular oxygen consumption rate in platelet mitochondria was measured using a filter-based multi-mode microplate reader (FLUOstar OPTIMA; BMG Labtech, Ofenburg, Germany) using MitoXpress-Xtra HS kits (Luxcel Company, Cork, Ireland) according to the manufacturer’s instructions. Fluorescence intensity was measured at 5 min intervals for 30 min at 37 °C in a sealed environment by applying 100 μL of mineral oil to reduce oxygen exchange. Tyrode’s buffer with 10 μL of MitoXpress-Xtra HS compound was used as reference. Each sample was analyzed in duplicate. The rate of fluorescence signal change per minute was calculated for each 5-minute interval, and the average signal change per minute over the entire 30-minute period was calculated for each sample.

### 4.6. Statistical Analysis

The results were subjected to one-way analysis of variance (ANOVA), using STATGRAPHICS Centurion XVI ver. 16.1.03 software. Arithmetic means and standard errors of the mean (SEMs) were calculated using the aforementioned program. The significance of the differences among groups was verified with Tukey’s test (HSD). The level of significance was set at *p* < 0.05, and data were presented as means ± standard errors of the mean (SEMs) (*n* = 8 for each group).

## 5. Conclusions

Our research showed a positive effect of CBD and nano-Se on the microstructure of the chicken breast muscle after *C. perfringens* infection. After adding these substances, an increase in the number of capillaries supplying individual muscle fibers was observed. The improvement in blood supply could have reduced degenerative changes in the muscles, i.e., fiber necrosis and splitting. This finding also indicated that a potential link between the condition of gut mucosa and histopathological changes in superficial pectoral muscle under induced inflammation may exist, and in this regard, CBD and nano-Se manifest ameliorative effects due to their protection against mucosal DNA damage. Further research is needed in this area to elucidate the mechanisms of action of the tested substances on skeletal muscles.

## Figures and Tables

**Table 1:** Effects of dietary treatments and *Clostridium perfringens* challenge on the microstructure of the superficial breast muscle in 35-day-old broiler chickens.

Trait	Group				
	CON	CON Positive	CBD	Nano-Se	CBD + Nano-Se
Number of capillaries per muscle fiber	0.97 ^b^ ± 0.07	0.98 ^b^ ± 0.05	1.20 ^ab^ ± 0.10	1.11 ^ab^ ± 0.09	1.31 ^a^ ± 0.12
Number of muscle fibers/1.5 mm^2^	165.38 ± 5.89	177.75 ± 12.70	165.13 ± 10.07	172.88 ± 7.82	178.13 ± 12.67
Muscle fiber diameter (μm)	48.56 ± 2.99	44.95 ± 5.62	47.59 ± 5.60	48.29 ± 3.10	45.66 ± 6.09
Number of necrotic fibers/1.5 mm^2^	1.63 ^ab^ ± 0.32	2.38 ^a^ ± 0.53	1.50 ^ab^ ± 0.60	0.38 ^b^ ± 0.26	1.00 ^b^ ± 0.38
Number of atrophic fibers/1.5 mm^2^	3.13 ^a^ ± 0.79	2.50 ^ab^ ± 0.53	1.13 ^b^ ± 0.30	2.25 ^ab^ ± 0.60	2.50 ^ab^ ± 0.42
Number of split fibers /1.5 mm^2^	2.00 ^b^ ± 0.65	4.13 ^a^ ± 0.55	1.88 ^b^ ± 0.40	1.63 ^b^ ± 0.60	1.13 ^b^ ± 0.40
Number of giant fibers/1.5 mm^2^	0.50 ± 0.27	0.63 ± 0.32	0.63 ± 0.32	0.75 ± 0.25	0.25 ± 0.16

CON—untreated control group; CON positive—birds fed a CON diet and challenged with *Clostridium perfringens* on days 15, 16, 17 and 18 of age; CBD—CON positive + dietary supplementation with cannabidiol; Nano-Se—CON positive + dietary supplementation with nano form of selenium; CBD+Nano-Se—CON positive + dietary supplementation with both additives. ^ab^—statistically significant differences at *p <* 0.05.

**Table 2:** Dietary treatments applied in the chicken experiments.

Treatment	CON	CON Positive	CBD	Nano-Se	CBD+Nano-Se
Commercial diet for broiler Ross 308 with no additives	+	+	+	+	+
*C. perfringens* Challenge	−	+	+	+	+
Cannabidiol	−	−	+	−	+
Nanosized selenium	−	−	−	+	+

CON—untreated control group; CON positive—birds fed a CON diet and challenged with *Clostridium perfringens* on days 15, 16, 17 and 18 of age; CBD—CON positive + dietary supplementation with cannabidiol; Nano-Se—CON positive + dietary supplementation with nano form of selenium; CBD+Nano-Se—CON positive + dietary supplementation with both additives.

## Data Availability

All data generated during the study are available from the corresponding author upon reasonable request.
